# The mechanism of degradation of alizarin red by a white-rot fungus *Trametes gibbosa*

**DOI:** 10.1186/s12896-021-00720-8

**Published:** 2021-11-05

**Authors:** Jian Zhang, Yujie Chi, Lianrong Feng

**Affiliations:** 1 School of Forestry, Northeast Forestry University, Harbin, 150040 China; 2 Liaoning Provincial Institute of Poplar, Gaizhou, 115213 Liaoning China

**Keywords:** Transcriptome, Alizarin red degradation, White fungi, LC–MS, GC–MS

## Abstract

**Background:**

Alizarin red (AR) is a typical anthraquinone dye, and the resulting wastewater is toxic and difficult to remove. A study showed that the white rot fungus *Trametes gibbosa* (*T. gibbosa*) can degrade dye wastewater by decolorization and has its own enzyme-producing traits.

**Methods:**

In this study, transcriptome sequencing was performed after alizarin red treatment for 0, 3, 7, 10, and 14 h. The key pathways and key enzymes involved in alizarin red degradation were found to be through the analysis of KEGG and GO. The Glutathione S-transferase (GST), manganese peroxidase (MnP) and laccase activities of *T. gibbosa* treated with alizarin red for 0–14 h were detected. LC–MS and GC–MS analyses of alizarin red decomposition products after 7 h and 14 h were performed.

**Results:**

The glutathione metabolic pathway ko00480, and the key enzymes GST, MnP, laccase and CYP450 were selected. Most of the genes encoding these enzymes were upregulated under alizarin red conditions. The GST activity increased 1.8 times from 117.55 U/mg prot at 0 h to 217.03 U/mg prot at 14 h. The MnP activity increased 2.9 times from 6.45 to 18.55 U/L. The laccase activity increased 3.7 times from 7.22 to 27.28 U/L. Analysis of the alizarin red decolourization rate showed that the decolourization rate at 14 h reached 20.21%. The main degradation intermediates were found to be 1,4-butene diacid, phthalic acid, 1,1-diphenylethylene, 9,10-dihydroanthracene, 1,2-naphthalene dicarboxylic acid, bisphenol, benzophenol-5,2-butene, acrylaldehyde, and 1-butylene, and the degradation process of AR was inferred. Overall, 1,4-butene diacid is the most important intermediate product produced by AR degradation.

**Conclusions:**

The glutathione metabolic pathway was the key pathway for AR degradation. GST, MnP, laccase and CYP450 were the key enzymes for AR degradation. 1,4-butene diacid is the most important intermediate product. This study explored the process of AR biodegradation at the molecular and biochemical levels and provided a theoretical basis for its application in practical production.

**Supplementary Information:**

The online version contains supplementary material available at 10.1186/s12896-021-00720-8.

## Background

Anthraquinone dyes are mostly aromatic compounds with stable conjugated structures, good light resistance and potential toxicity. Because of the special properties of anthraquinone dyes, the wastewater produced is not only high in organic composition but also has high chromaticity, complex composition and toxicity, it is difficult to oxidize and to degrade by chemical methods. It is easy to dissolve AR in water and difficult to remove it by conventional physical methods, which brings some difficulty to wastewater treatment, and affects the ecological environment [1–3]. As an anthraquinone dye, it is often representative of the degradation of dye wastewater. Alizarin Red (AR, formula: C_14_H_7_NaO_7_S·H_2_O), also known as 9,10-dihydro-3,4-dihydroxy-9,10-dioxo-2-anthracenesulfonic, aci monosodium s, is a yellowish-brown or orange dye occurring in powder form obtained via Alizarin sulfonation. It is widely used as an acid–base indicator and a dye. When used as a textile industry stain, approximately 10–15% of the dye is lost into water to form dye wastewater. AR can be inserted between the base pairs of the DNA double helix, thus doubling its oxidative damage efficiency. Therefore, AR should be treated before being discharged into water. The color-rendering structure of AR is an anthraquinone ring. Hence, it is necessary to destroy the quinone ring structure first to degrade AR. The degradation methods of anthraquinone dye wastewater mainly include physical, chemical and biological methods. The chemical treatment of dyes easily causes secondary pollution, and physical bleaching has the disadvantages of high cost and low efficiency [4]; however, the biological treatment of dye wastewater is efficient and without secondary pollution [5]. According to previous research, white rot fungi have certain decolorization and degradation abilities in dye wastewater [6]. White rot fungi secrete a variety of intracellular and extracellular enzymes, such as extracellular manganese peroxidase (MnP), lignin peroxidase (LiP) and laccase [7–10], and intracellular cytochrome P450 (CYP450) [11, 12]. The extracellular oxidase produced in secondary metabolism can degrade all kinds of dyes in a broad spectrum, especially for many synthetic compounds such as polycyclic aromatic hydrocarbons (PAHs) and polychlorinated biphenyl compounds. However, the mechanism of dye degradation by white rot fungi is not well studied. Using transcriptome technology to study various biological phenomena at the molecular level is a popular research method [13].

On the basis of previous studies, HiSeq high-throughput sequencing technology was used to study the changes in the transcription level of *Trametes gibbosa* (*T. gibbosa*) treated at 0, 3, 7, 10, and 14 h in an anthraquinone dye alizarin red environment. To explore the key pathways, genes and their regulated enzymes of *T. gibbosa* involved in alizarin red dye degradation were studied. Enzyme activity related to alizarin red degradation was detected. AR degradation intermediate products were analysed by LC–MS and GC–MS to explore the degradation pathway of AR. For white rot fungi, the mechanism of *T. gibbosa* degradation of anthraquinone dye AR provides molecular-level data support and a theoretical basis.

## Materials and methods

### Fungal culture and the hyphal collection

Source of materials: Our limited collection of the *T. gibbosa* CB1 fruiting bodies for research is allowed. The strain was collected by Professor Chi Yujie in Changbai Mountain, Jilin Province, China, and then underwent tissue separation in the laboratory. Store in the Forest Pathology Laboratory of School of Forestry, Northeast Forestry University. Sampling site is located in Changbai Mountain Nature Reserve, Northeast China (12,742′55″–12,816′48″ E, 4141′49″–4251′18″ N) with an average altitude of 802 m. Professor Chi Yujie and Doctor Yan Hongbo sequenced the collected subentities and the isolated mycelium extract DNA for ITS, and the PCR product sequencing of the rDNA-ITS region of the strain CB1 obtained a 601 bp nucleotide sequence. Submit this sequence to NCBI's GenBank (NCBI Accession No: JF279440).

The *T. gibbosa* CB1 preserved strain was inoculated on PDA plate medium (9 cm) and cultured at 25 °C for 7 days and to obtain hyphae from the edge of the plate with a 5 mm^2^ punch. Hyphae were inoculated into a conical flask (250 mL) containing 5 ml filtered and sterilized 15% glucose (V/V) and 70 mL LNAS medium. The formulation of LNAS medium as follow:

LNAS (Low Nitrogen Asparagine Succinate): KH_2_PO_4_ 0.2 g, MgSO_4_·7H_2_O 0.5 g, Succinic acid 1.18 g, CaCl_2_·2H_2_O 0.1 g, mineral solution 1 mL, nitrogen solution 19.4 mL, vitamin solution 0.5 mL, pH = 4.5.

Mineral solution (L^−1^): Ferric-citrateH_2_O 0.087 g, ZnSO_4_·7H_2_O 0.1 g, MnSO_4_·H_2_O 0.45 g, CoCl_2_·6H_2_O 0.1 g, CuSO_4_·5H_2_O 0.01 g.

Nitrogen solution (L^−1^): l-asparagine·H_2_O 4.0 g, NH_4_NO_3_ 2.0 g.

Vitamin solution (L^−1^): d-biotin 0.002 g, Folic acid 0.002 g, Thiamine·HCl 0.005 g, Riboflavin (VB2) 0.005 g, Pyridoxine·HCl (VB6) 0.01 g, Cyanocobalamin (VB12) 0.0001 g, Nicotinic acid (Vpp) 0.005 g, Calsium Pantothenate (VB3) 0.005 g, P-aminobenzoic acid 0.005 g, DL-6, 8-thioctic acid 0.005 g.

After static culture at 25 °C for 10 days, alizarin red (AR, C_14_H_7_NaO_7_S·H_2_O) dye was added to each bottle, and the final concentration of AR dye in the medium was 50 mg/L. Alizarin red was shaken and mixed into LNAS. Hyphae were extracted after 0 h, 3 h, 7 h, 10 h and 14 h of AR treatment. Shake well manually every 30 min. Among them, 0 h was set as the control group (no dye treatment) mark as CK, and the others were the experimental group, mark as QSH1, 2, 3 and 4. Each group had three biological repetitions, each biological repetition consisting of 5 bottles of treated hyphae. After mixing the hyphae and then placed into the frozen storage tube, sealed, put into liquid nitrogen for freezing, and then put into the − 80 °C refrigerator and saved for future analysis.

### RNA extraction, cDNA library sequencing of the transcriptome, and the assembly and splicing of sequences

Frozen hyphal samples were sent to Beijing BMK Biotechnology Co., Ltd. for transcriptome sequencing by Illumina HiSeq X Ten high-throughput sequencing platform. Data were analysed after quality control of raw data obtained by sequencing (clean reads). Sequence alignment of clean reads with JGI *T. gibbosa* reference genomes using software. The genome was published in December 2016. The resulting mapped data and Cufflinks were compared with mapped data reads. The transcripts were assembled according to the GFF documents of the reference genome, and the Cuffmerg transcripts were merged to obtain a complete set of transcript information and the expression value of each gene. Using BLAST [14] software, the genes obtained from the transcriptome were functionally annotated in the COG, eggNOG, GO, KEGG, NR, and SwissProt gene functional annotation databases.

### Analysis of gene differential expression in AR

BMKCloud (www.biocloud.net) and edgeR software (https://bioconductor.org/packages/release/bioc/html/edgeR.html) were used, and FDR = 0.01 and FC = 2 were selected as the DEG screening criteria [15]. Analysis of differential expression was performed in the following groups: CK versus QSH1; CK versus QSH2; CK versus QSH3; CK versus QSH4; QSH1 versus QSH2; QSH1 versus QSH3; QSH1 versus QSH4; QSH2 versus QSH3; QSH2 versus QSH3; QSH3 versus QSH4. The differentially expressed gene (DEG) sets between the groups were obtained. Then, the total number of DEGs was obtained, and GO and KEGG analyses of DEGs were performed.

### Detection of AR decolorization rate

The maximum absorption peak and its wavelength were determined by scanning 50 mg/L AR between 300 and 800 nm with a spectrophotometer. The absorbance value of AR was measured at wavelength at the maximum absorption peak with the average of 3 sets of data, according to the absorption value of the test group minus the absorption value of the control group, as Ai and the absorptivity of the 50 mg/L AR minus the absorptivity of the control group as A_0_. The decolorization rate was calculated by the following formula.

$${\text{Decolourization\,rate\,(\%)}}= \frac{{{\text{A0}} - {\text{Ai}}}}{{{\text{A0}}}} \times 100\%$$

### Detection of extracellular enzyme MnP and laccase activity

The extraction of 3 mL of culture medium was performed in 5 stages (three replicates per group), followed by 13,000 r/min centrifugation for 10 min, and extraction of the supernatant. Extracellular enzyme Laccase and MnP activity related to AR degradation were detected. Laccase and MnP activity detection methods as follow [16, 17].

Laccase activity detection methods: Determination system 1 mL. Sodium propionate buffer (50 mmol L^−1^, pH = 4.5) 850 μL, 2; 2′-azino-bis (3-ethylbenzthiazoline-6-sulfonic acid) (ABTS 20 mmol L^−1^) 50 μL, Enzyme liquid sample 50 μL, H_2_O 50 μL. 1 mL deionized water as control. The change of absorbance OD value in 3 min at 420 nm is used as the Laccase activity value.

MnP activity detection methods: Determination system 1 mL. Sodium propionate buffer (50 mmol L^−1^, pH = 4.5) 840 μL, MnSO_4_ (10 mmol L^−1^) 50 μL, 2,6-DNP (10 mmol L^−1^) 50 μL, Enzyme liquid sample 50 μL, H_2_O_2_(10 mmol L^−1^) 10 μL. 1 mL deionized water as control. The change of absorbance OD value in 1 min at 470 nm is used as the MnP activity value.

Formula for calculating enzyme activity:${\text{Enzyme \, activity}}\,(U/L) = \frac{\Delta OD}{{\Delta t}} \times \frac{Vtotal}{{{\text{Ve}}}} \times \frac{{10^{6} }}{\varepsilon } \times {\text{N}}$.

In the formula:V_total_ is total volume of reaction; Ve is volume of the enzyme solution; N is dilution multiple of enzyme solution; ε is molar extinction coefficient, ε420 = 36 000 mol L^−1^ cm^−1^, ε470 = 49 600 mol L^−1^ cm^−1^; Δt is reaction time; ΔOD is incremental value of absorbance change during reaction time.

### Detection of intracellular GSH content and intracellular GST activity

*T. gibbosa* hyphal treatment was extracted by AR at 5 stages and rinsed and dried in phosphate-buffered saline (PBS). Hyphae were ground into powder with liquid nitrogen, and then an equal proportion of PBS homogenate was used. Then, the samples were centrifuged to extract the supernatant and set aside. Glutathione S-transferase (GST), Glutathione (GSH) and the Total protein (TP) assay kit (Cargo Number: A004: http://www.njjcbio.com/uploadfile/product/big/20190805111314152.pdf, A006-1–1: http://www.njjcbio.com/uploadfile/product/big/20190805111415011.pdf and A045-2: http://www.njjcbio.com/uploadfile/product/big/20190807092324790.pdf, Nanjing Jiancheng Bioengineering Institute, China) methods and formulas were used to detect the GST and GSH activity of *T. gibbosa* mycelium at each stage.

### LC–MS and GC–MS were used to detect decomposition products AR at different time points

Degradation of dye molecules during the *T. gibbosa* decolourization process passes through some intermediate products that were analysed by LC–MS and GC–MS and identified by interpretation of their mass spectral data presenting their molecular ion peaks with respect to m/z (where m is the molecular weight of the intermediates in the mass spectra). Thus, the molecular cleavage pathway of the AR dye is deduced. The *T. gibbosa* culture medium (100 mL) was extracted after AR treatment for 7 and 14 h, and the culture medium was pre-treated.

The sample pre-treatment of culture medium was as follows: Dichloromethane were taken and activated in the C18 column stationary phase. Methanol was taken through the column to remove impurities. Finally, the column was washed with deionized water. The culture medium over the C18 column and mixture solution were prepared using 2 mL methanol and 8 mL dichloromethane, and the intermediate product was eluted from the C18 column and coexisted in the test tube.

Descriptions of various test parameters and data processing procedure are found below.

1. LC–MS test parameters: Chromatographic column: Agilent SB-C18RRHD, 1.8 μm, 2.1*100 mm, column temperature: 35 °C. Mobile phase: A 5 mM ammonium acetate solution, B methanol. Flow rate: 0.3 mL/min. Injection:5μL. Automatic sampler temperature/TEM: 25℃, DAD detection wave: 519 nm.
  Mass Spectrometry Acquisition Parameters: Ion source type: ESI, Pattern: MS2 Scan, Polarity: neg, m/z range: 100–370, Ion source temperature:350℃.
2. GC–MS test parameters: Sampler: 7683B, The mass spectrometry used an EI ion source, electron energy of 70 eV, scanning range of 50–500, four-stage rod temperature of 150 °C, ion source temperature of 230 °C, and transmission line temperature of 280 °C. For related parameters see Table 1.

**Table 1:** The relevant parameters of LC–MS operation

Controlling factor	Parameter conditions	Controlling factor	Parameter conditions
Maximum scanning speed	10.0 mAu/s	Carrier gas	He
Detector	MSD	Sample size	10 μL

### Results

#### Results of transcriptome data and quality control evaluation

**Fig. 1:**
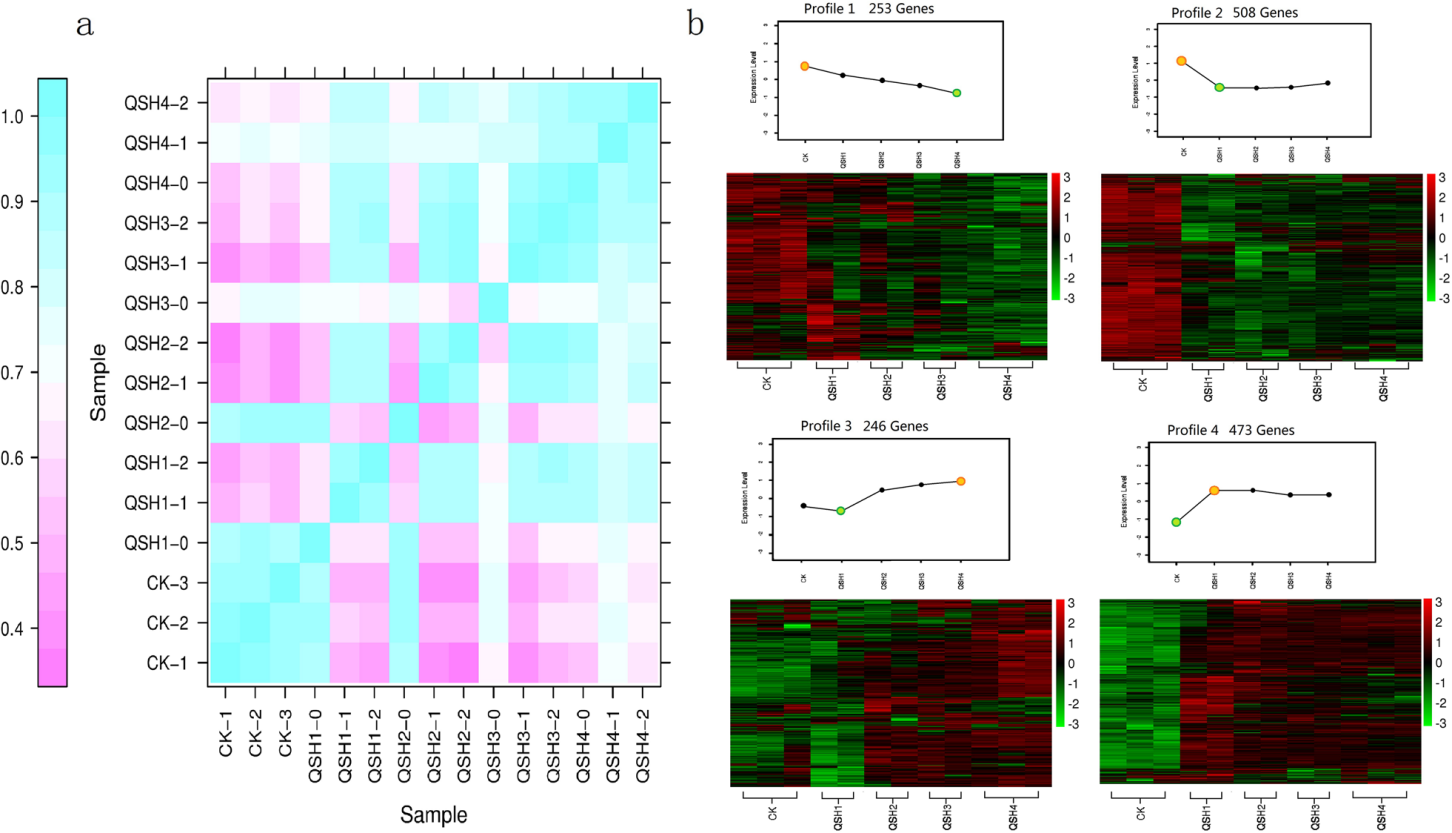
**a** RNA-seq correlation detection. Rows and columns are all 15 sample names, and the intersection of rows and columns is the square of the correlation coefficient between samples (Pearson correlation coefficient square, R2). The larger the value is, the stronger the correlation is. **b** Gene expression patterns at five time points in the AR environment. Yellow indicates the highest expression of this pattern, and green indicates the lowest level

A total of 109.29 Gb clean data were obtained from transcriptome analysis of 15 samples, the percentage of Q30 bases was 91.32% and above, and the quality of transcriptome data was up to standard. is the clean reads of each sample were compared with the designated reference genome, and the alignment efficiency was 87.22–91.32%, and reference genome selection was correct and efficient (Additional file 1: Table S1). A total of 12,921 genes were obtained of which 10,560 genes were annotated, and the annotation efficiency was 81.72%. There were 883 new genes, 162 of which were annotated (Additional file 1: Table S2). Three of the 15 samples (QSH1-0, QSH2-0, and QSH3-0) were significant outliers compared with the other 2 samples in the same group. Except for these 3 outlier samples, the other 12 samples showed good correlation (Fig. 1a).

### Gene Expression Pattern Analysis and Clustering of DEGs

The transcriptome data were divided into 10 groups, among which 6 groups had more downregulated genes than upregulated genes (Additional file 1: Table S3). Ten differential groups received a total of 1480 DEGs of which 1370 DEGs were annotated. A total of 4 expression trends were obtained by analysing 1480 DEG expression trends (Fig. 1b). Profile 1 and Profile 2 genes were downregulated, for a total of 761 genes. Profile 3 and Profile 4 genes belonged to the upregulation type, for a total of 719 genes. There were more downregulated genes than upregulated genes. The expression of Profile 4 genes increased at 0–3 h and decreased slightly at 3–14 h. Profile 1 and Profile 2 genes were inhibited after exposure to AR stimulation and stress. The expression of the Profile 3 and Profile 4 genes increased after AR induction.

According to the differential trend analysis of DEGs, it was found that for a total of 66.3% of DEGs (Profile 2 and Profile 4) at 0–3 h were the most variable stages of DEG expression, 3–14 h expression was stable, and 17.1% of DEGs (Profile 1) continued to change at 0–14 h. The expression of 16.6% of DEGs (Profile 3) at 0–3 h was stable, and the expression changed greatly at 3–14 h, and 0 h, 3 h and 14 h are important nodes for DEG expression. The expression of all DEGs can be divided into two stages: 0–3 h and 3–14 h. Therefore, the analysis of CK versus QSH1 (0 h vs. 3 h) and QSH1 versus QSH4 (3 h vs. 14 h) were more representative of the role of *T. gibbosa* DEGs in AR degradation.

### Differential grouping of DEGs in the GO database analysis

**Fig. 2:**
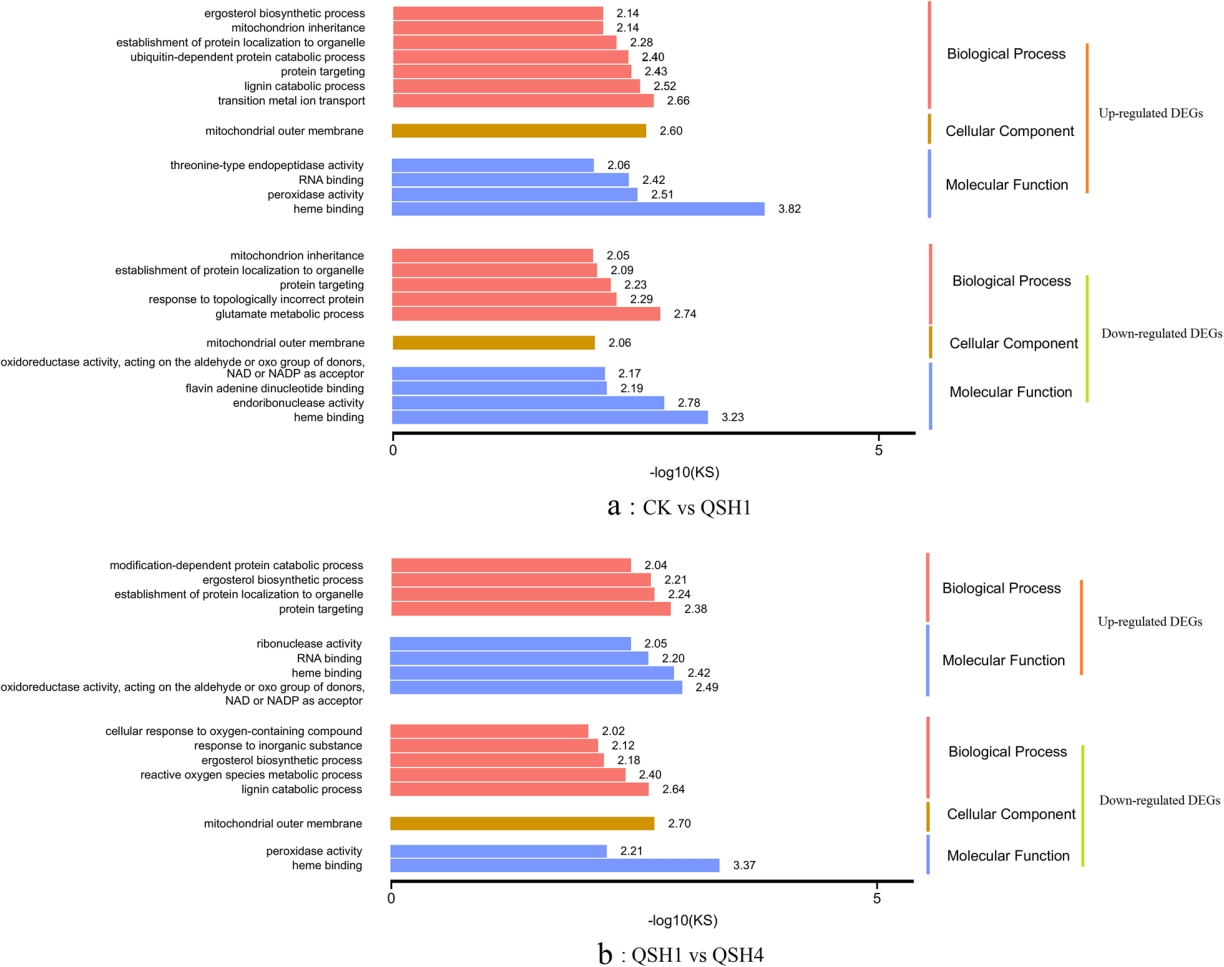
Enrichment analysis GO to DEGs. **a** GO enrichment of CK versus QSH1 DEGs. **b** GO enrichment of QSH1 versus QSH4 DEGs

The DEGs of the different groups CK versus QSH1 and QSH1 versus QSH4 were GO enriched. The enrichment categories of log10(KS) ≥ 2 are intercepted (Additional file 1: Table S4). KS: the significant statistics of the enrichment of the GO category, the smaller the KS value is the larger the log10(KS) is, indicating the more significant the enrichment. At 0–3 h, upregulated DEGs were significantly enriched to GO:0,020,037 (heme binding), GO:0,000,041 (transition metal ion transport), GO:0,005,741 (mitochondrial outer membrane), GO:0,046,274 (lignin catabolic process) and GO:0,004,601 (peroxidase activity) categories, and downregulated DEGs were significantly enriched to GO:0,020,037 (heme binding), GO:0,004,521 (endoribonuclease activity) and GO:0,006,536 (glutamate metabolic process) categories (Fig. 2a). At 3–14 h, upregulated DEGs were significantly enriched in the GO:0,016,620 (oxidoreductase activity, acting on the aldehyde or oxo group of donors, and NAD or NADP as the acceptor) and GO:0,020,037 (heme binding) categories, and downregulated DEGs were significantly enriched in the haem binding, lignin catabolic process and reactive oxygen species metabolic process categories (Fig. 2b). Four important categories were enriched in both phases: GO: 0,020,037 (heme binding), GO: 0,046,274 (lignin catabolic process), GO: 0,016,620 (oxidoreduction activity, acting on the aldehyde or oxo group of donors, and NAD or NADP as the acceptor) and GO: 0,004,601 (period activity). Haem is the cofactor of haemoglobin and myoglobin, cytochrome, peroxidase, and catalase, which controls the synthesis and expression of redox enzymes such as the MnP haem-binding category, which can be determined to be associated with redox reactions. Lignin is a polymer in which phenylpropanoid structural units are irregularly coupled by ether and carbon bonds [18]. However, most dyes are similar to lignin structures and are composed of heterocycles and aromatic rings, all of which are aromatic compounds. Therefore, the significant enrichment of the lignin catabolic process class can be determined to be related to AR degradation.

Based on two stages of GO enrichment analysis, at 0–3 h, *T. gibbosa* secondary metabolic activity and redox reactions become active, and basic life activities such as carbohydrate utilization and protein synthesis are inhibited. At approximately 14 h, the redox reaction activity decreased relative to that at 3 h and carbohydrate transport and metabolism as well as energy production and transformation functions at 14 h.

All 1480 DEGs were compared to the four important categories enriched above (Table 2). The 20 DEGs enriched in haem-binding classes encoded 8 enzymes: 4-hydroxysphinganine ceramide fatty acyl 2-hydroxylase, Versatile peroxidase, MnP, Acyl-CoA dehydrogenase, Acyl-CoA desaturase, Cytochrome P450 (CYP450), Fumarate reductase, L-lactate dehydrogenase (cytochrome) and nitric oxide dioxygenase. Laccase of all 3 DEGs was enriched in the lignin catabolic process category. Two DEGs were enriched in oxidoreductase activity, acting on the aldehyde or oxo group of donors, NAD or NADP as acceptor categories, encoded 1-pyrroline-5-carboxylate dehydrogenase and aldehyde dehydrogenase (NAD). Peroxidase activity of four DEGs encoded Versatile peroxidase (VP) and MnP genes, and all of them were redox enzymes. Among them, MnP and VP appear in two categories. The CYP450 enrichment genes were the most. Laccase-enriched functional categories were most correlated with AR degradation. According to the GO enrichment analysis, the redox reaction plays a role in AR degradation. MnP, VP, CYP450 and laccase are key enzymes for the redox decomposition of AR.

**Table 2:** DEGs from four important GO terms

GO term	Gene function	Gene no
Heme binding	4-Hydroxysphinganine ceramide fatty acyl 2-Hydroxylase	gene_5570
	Versatile peroxidase	gene_11851, _11537, _713
	MnP	gene_8611
	Acyl-CoA dehydrogenase	gene_22, _4
	Cytochrome P450	gene_7628,_9113,_4488,_5216,_11472,_4487, gene_5139,_7522,_10935,_6568
	Fumarate reductase	gene_8119
	l-Lactate dehydrogenase (cytochrome)	gene_4787
	Nitric oxide dioxygenase	gene_10651
Lignin catabolic	Laccase	gene_3889,_3902,_1741
Oxidoreductase activity, acting on the aldehyde or oxo group of donors, NAD or NADP as the acceptor	1-PYRROLINE-5-carboxylate dehydrogenase	gene_1202
	Aldehyde dehydrogenase (NAD +)	gene_3674
Peroxidase activity	Versatile peroxidase	gene_11851,_11537, _713
	MnP	gene_8611

### Differential grouping DEG KEGG metabolic pathway analysis

**Fig. 3:**
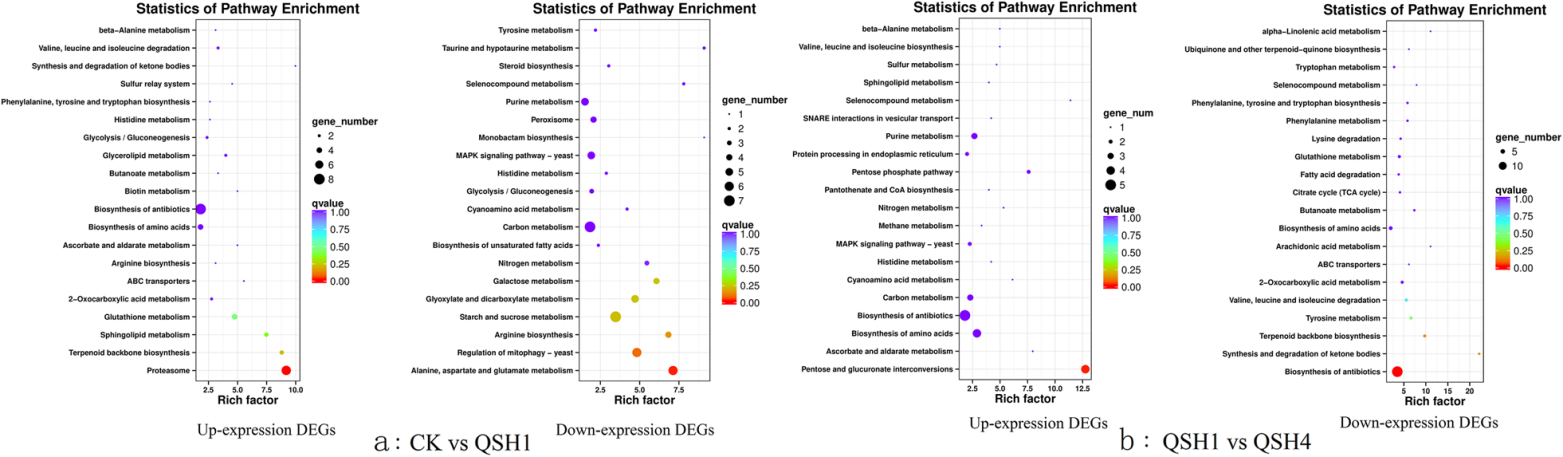
Enrichment analysis KEGG to DEGs. **a** KEGG enrichment of CK versus QSH1 DEGs. **b** KEGG enrichment of QSH1 versus QSH4 DEGs

The DEGs of CK versus QSH1 (0 h vs. 3 h) and QSH1 versus QSH4 (3 h vs. 14 h) were KEGG enriched, rich factor ≥ 4, and q value ≥ 1. Upregulated DEGs at 0–3 h were significantly enriched in the proteasome, terpenoid backbone biosynthesis, sphingolipid metabolism and glutathione metabolism pathways. Downregulated DEGs at 0–3 h were significantly enriched in alanine, aspartate and glutamate metabolism, regulation of mitophagy—yeast, arginine biosynthesis, starch and sucrose metabolism, glyoxylate and dicarboxylate metabolism, galactose metabolism and the nitrogen metabolism pathways (Fig. 3a).

Upregulated DEGs at 3–14 h were significantly enriched in the pentose and glucuronate interconversion pathways. Downregulated DEGs at 3–14 h were enriched in biosynthesis of antibiotics, synthesis and degradation of ketone bodies, terpenoid backbone biosynthesis, tyrosine metabolism and valine, leucine and isoleucine degradation (Fig. 3b).

According to DEG enrichment of KEGG pathways at 0–3 h, genes involved in glutathione metabolism expression were upregulated by AR at this stage. Downregulation of the gene enrichment pathway found that the addition of AR affected the metabolism of substances needed for basic life activities such as glucose metabolism and energy metabolism in *T. gibbosa*. At the 3–14 h stage, glucose metabolism genes began to be upregulated, and the metabolism of amino acids and lipids was downregulated. Terpenoid synthesis gene transcription began to be downregulated.

Glutathione metabolism and redox reactions are the main processes involved in AR degradation by KEGG and GO database analysis.

### Redox reactions and glutathione metabolism are involved in AR degradation

**Fig. 4:**
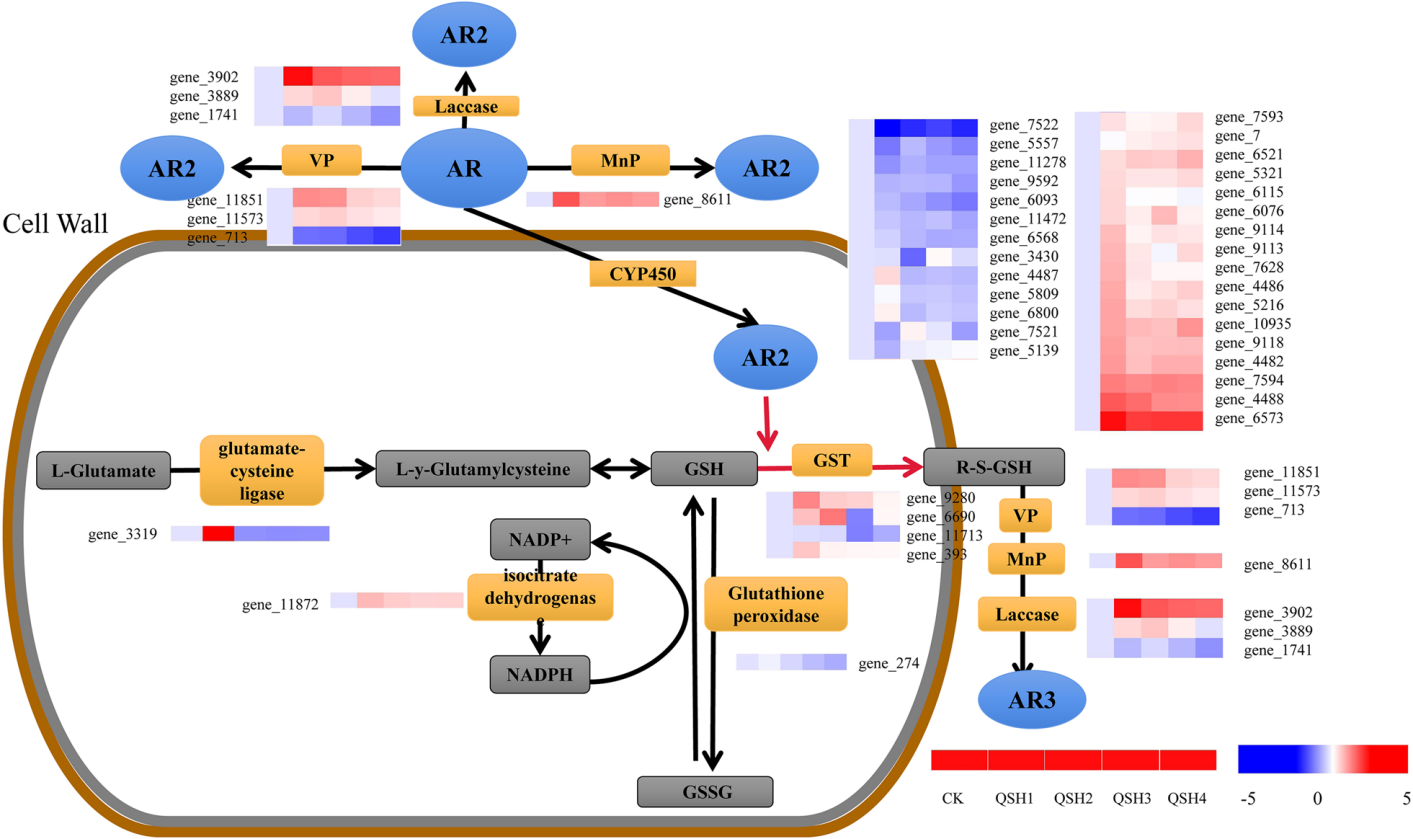
*L. gibbosa* DEG transcriptional changes in the AR metabolic pathway. The enzymes in response to AR are represented in orange boxes, whereas the other metabolites are in grey boxes. For enzyme reactions, the arrows between two metabolites represent the directions of catalytic reactions. The expression patterns over the five time points of the genes encoding corresponding enzyme(s) are given above or under the arrows (based on the CK expression of each gene, set to 0). AR2 and AR3 represent metabolite production of AR under enzymatic action

Some important reactive redox reactions and glutathione metabolism pathway related to AR degradation were identified by transcriptome analysis (Fig. 4). Exogenous chemicals are generally eliminated in two ways: one is discharged directly from the body without metabolism, and the other is excreted in the form of metabolites after metabolism. The metabolism of exogenous chemicals in vivo mainly undergoes a two-step reaction. The first step is the phase I reaction in which exogenous chemicals are oxidized, reduced or hydrolysed to more polar metabolites. The key enzyme to catalyse the phase I reaction is the CYP450 enzyme system. The second step is the phase II reaction in which exogenous chemicals and their metabolites are combined with endogenous substances and discharged in vitro. There are many enzymes that catalyse the phase II reaction. The important enzymes are glucuronic acid transferase, GST and N-phthalyl transferase. When AR enters *T. gibbosa*, it is first oxidized by CYP450 and other phase I enzymes. Then, bound to reduced glutathione (GSH) under GST catalysis it is excreted (this process is an important detoxification process in organisms). AR metabolites were treated in vitro with CYP450 and GST and then oxidized and decomposed by the extracellular enzyme laccase and MnP and other oxidases.

During this study, 1 MnP gene (gene_8611) was upregulated; three Laccase genes (gene_3902,_3889, and _1741) 2 upregulated and 1 downregulated.; three VP genes (gene_11851,_11573, and _713), 2 upregulated and 1 downregulated; 4 GST genes (gene_9280,_6690,_11173 and _393), 3 upregulated and 1 downregulated and 30 CYP450 genes (gene_7522, _5557, _11278, _9592, _6093, _11472, _6568, _3430, _4487, _5809, _6800, _7521, _5139, _7593, _7, _6521, _5321, _6115, _6076, _9114, _9113, _7628, _4486, _5216, _10935, _9118, _4482, _7594, _4488, and _6573), 23 had upregulated expression and 7 had downregulated expression. These inferences may be involved in most of the upregulation of genes encoding AR-degrading enzymes under AR conditions. The glutathione peroxidase gene and isocitrate dehydrogenase gene, of genes involved in GSH production, also showed differential expression and upregulated expression. The molecular level shows that MnP, laccase, CYP450, VP, GST and GSH are involved in AR degradation.

#### GST and GSH activity determination results

**Fig. 5:**
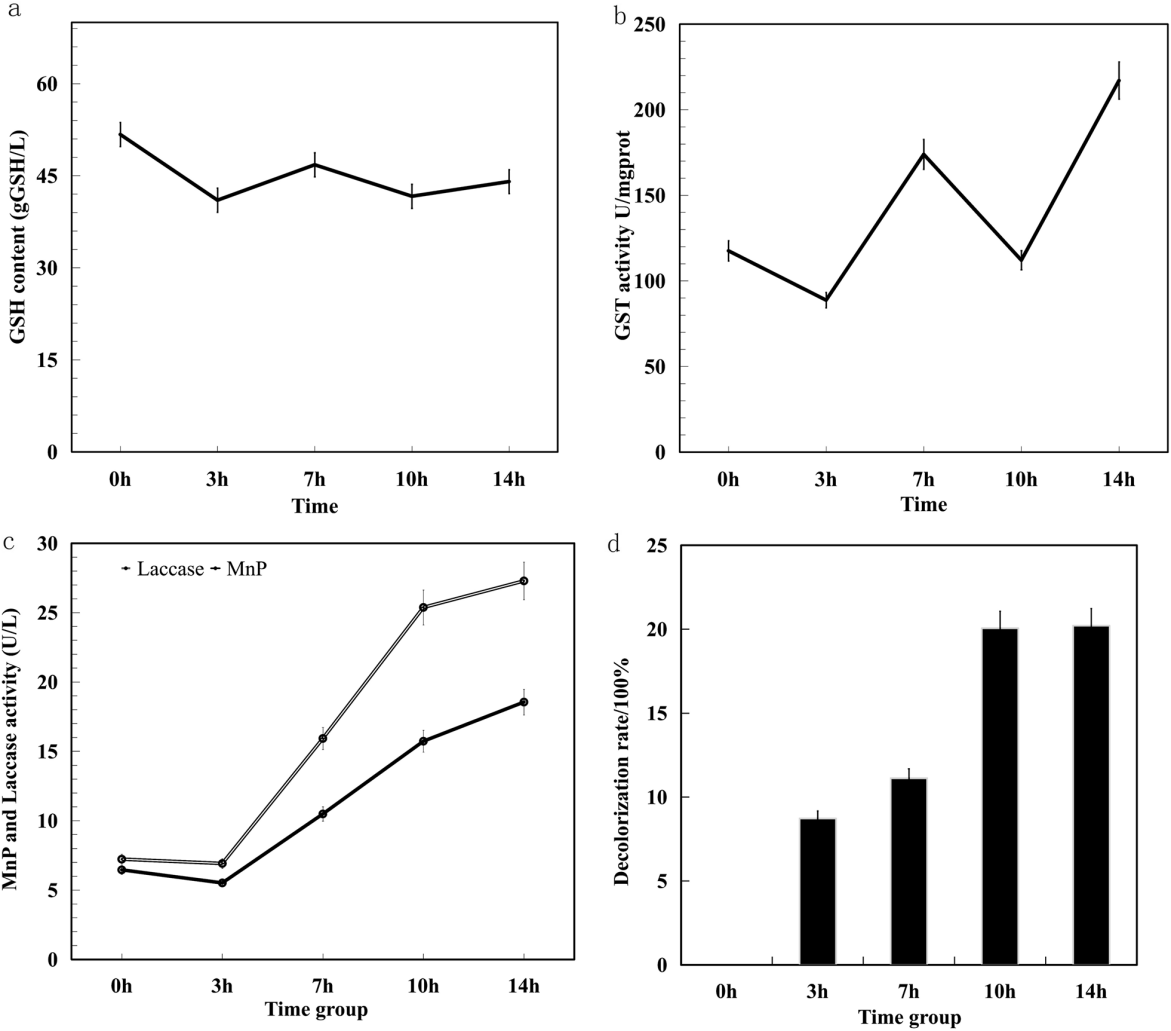
Detection of the activity of various substances of *T. gibbosa*. **a** GSH content. **b** GST activity **c** MnP and Laccase activity. **d** AR decolourization rate

To verify whether the GST and GSH responds to AR, the GST activity and GSH content of hyphae in the 5 treatment stages were detected. The GSH content decreased slightly from 0 h 51.71 gGSH/L to 14 h 44.04 gGSH/L (Fig. 5a). Down by 14.83%. The GST activity of 0–3 h decreased, 3–7 h increased sharply, 7–10 h decreased, and 10–14 h increased violently. GST activity showed an overall upward trend, from 0 h 117.55U/mg prot to 14 h 217.03U/mg prot, and enzyme activity increased 1.8 times (Fig. 5b). Figure 5a, b show that despite the overall trend of decrease in GSH, GST increased. However, the trends at various time nodes were consistent, with 0–3 h and 7–10 h being down and 3–7 h and 10–14 h being up, respectively. GST activity dramatically fluctuated over each time period; GSH exhibited the same trend as GST fluctuations, but the estimate of GSH activity exhibited only small variations. GST activity and GSH content showed that the GSH content slightly decreased GST, and a large amount of secretion promoted the binding of GSH and AR. Thus, glutathione metabolism responds to AR and participates in intracellular AR degradation.

#### Detection of redox enzyme activity participates in AR degradation

Extracellular enzyme laccase and MnP activity were determined at the 5 AR treatment stages. The results showed that laccase and MnP enzyme activities decreased slightly at 0–3 h, increased sharply at 3–10 h and then the upward trend at 10–14 h slowed down (Fig. 5c). Laccase activity increased 3.7 times from 0 h 7.22 U/L to 14 h 27.28 U/L. MnP activity from 0 h 6.45 U/L to 14 h 18.55 U/L, and enzyme activity increased 2.9 times. The results showed that with the increasing *T. gibbosa* time in the AR dye environment, the activities of laccase and MnP increased. At 3–10 h, activity increased most dramatically in both enzymes. These two enzymes respond to AR and act extracellularly.

### T. gibbosa Decolourization of AR

AR decolourization was determined at 0, 3, 7, 10, and 14 h (Fig. 5d). The decolourization rate increased at 0–10 h. By 10 h, the decolourization rate reached 20.06%. At 10–14 h, the decolourization rate was stable and unchanged. At 14 h, the final decolourization rate was 20.21%. The explanation for these findings is that *T. gibbosa* has a decolourization effect on AR.

The results of combined enzyme live assay revealed that the activity change trend of the extracellular enzyme laccase and MnP was consistent with the discoloration rate change trend of AR. Moreover, the above parameters significantly changed at 3–10 h and smoothly changed at 10–14 h. The activity of laccase and MnP was associated with the AR decolorization rate, while the activity of GSH and intracellular enzyme GST fluctuated, with no significant correlation with changes in the rate of dechromination.

### Inference of AR Decomposition

#### Analysis of AR degradation products by LC–MS and GC–MS techniques

**Fig. 6:**
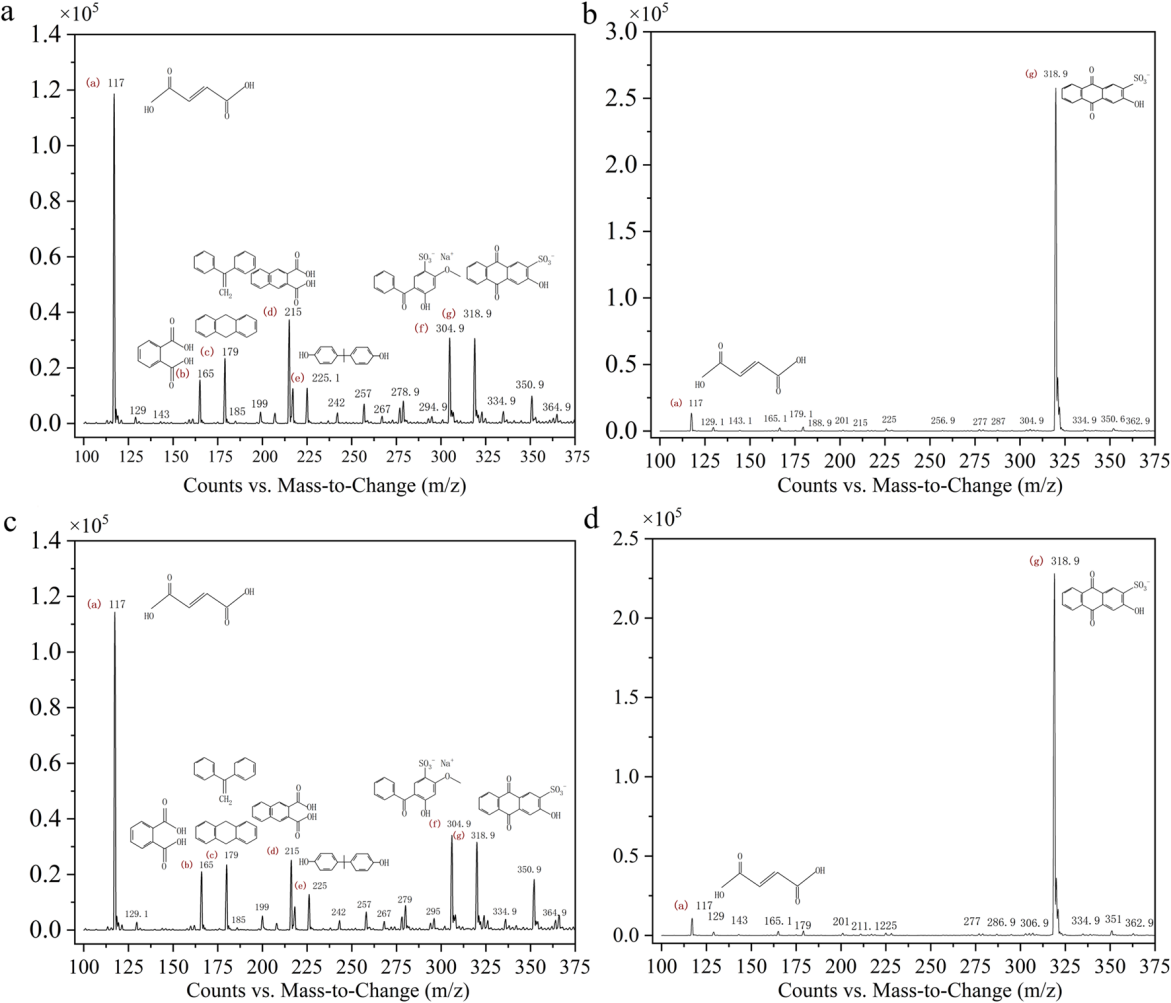
GC–MS mass spectrometry of AR degradation production by *T. gibbosa* at various stages. **a** and **b** The decomposition products of AR at 7 h were analysed by LC–MS, **c** and **d** 14 h production spectrum of AR

To explore the possible degradation pathways of AR, LC–MS was carried out by sampling the intermediates as well as the final and stable degraded products. The mass-to-load ratio (m/z) of particle fragments can be obtained by LC–MS. The results of LC–MS analysis showed that the types of intermediate products obtained at 7 h and 14 h were the same, but the contents of each substance at 14 h were obviously less than those at 7 h. Seven substances (Table 3; Fig. 6a–d) were obtained with mass-to-load ratios of 318.9, 117.1, 165.0, 179.1, 215.0, 225.0, and 304.9. On the basis of m/z, 318.9 is AR (alizarin red removal Na), and 304.9, 179.1, 225 and 165 are benzophenone-5, 1,1-diphenylethylene, bisphenol and phthalic acid, respectively. These four substances may be formed by AR from the benzene ring opening in the middle. The m/z 215.0 is 1,2-naphthalene dicarboxylic acid, and the carbonyl addition reaction may occur from AR and form ring openings from sulfite roots. The m/z 117.1 is a 1,4-butene diacid, which is a small molecular substance formed by cracking of the benzene ring. Figure 6a–d shows that 1,4-butene diacid is the most abundant AR decomposition intermediate and is the most important intermediate product of *T. gibbosa* degradation of AR. The m/z 179.1 can also be inferred to be 9,10-dihydroanthracene, which is derived from AR de-sodium ions, hydroxyl groups and sulfite ions. The anthraquinone structure of AR is destroyed, and the anthraquinone structure is the hair colour group of AR. The anthraquinone structure was destroyed, and the dye colour disappeared.

**Table 3:**
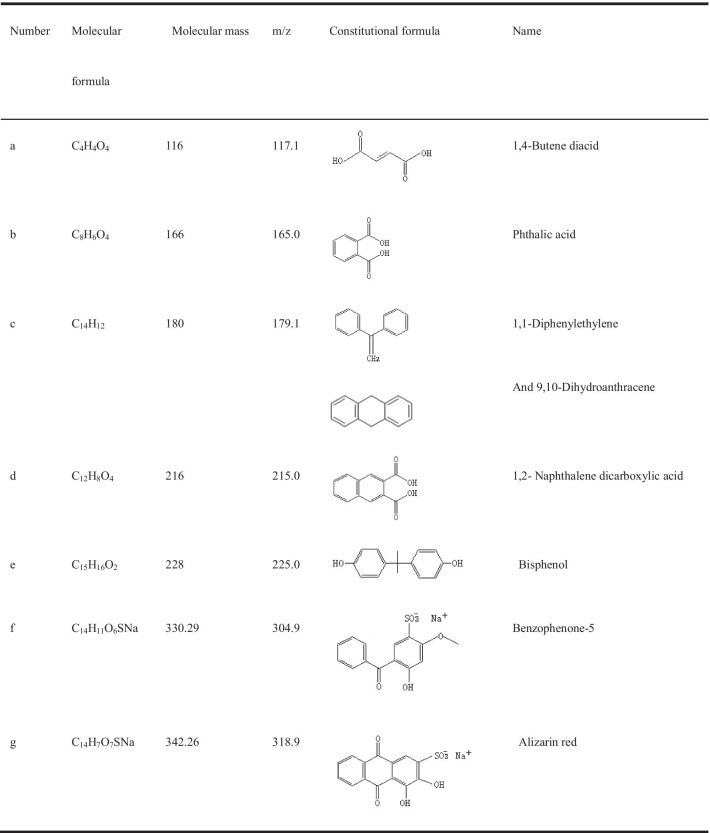
Relevant information of the intermediates by LC–MS

**Fig. 7:**
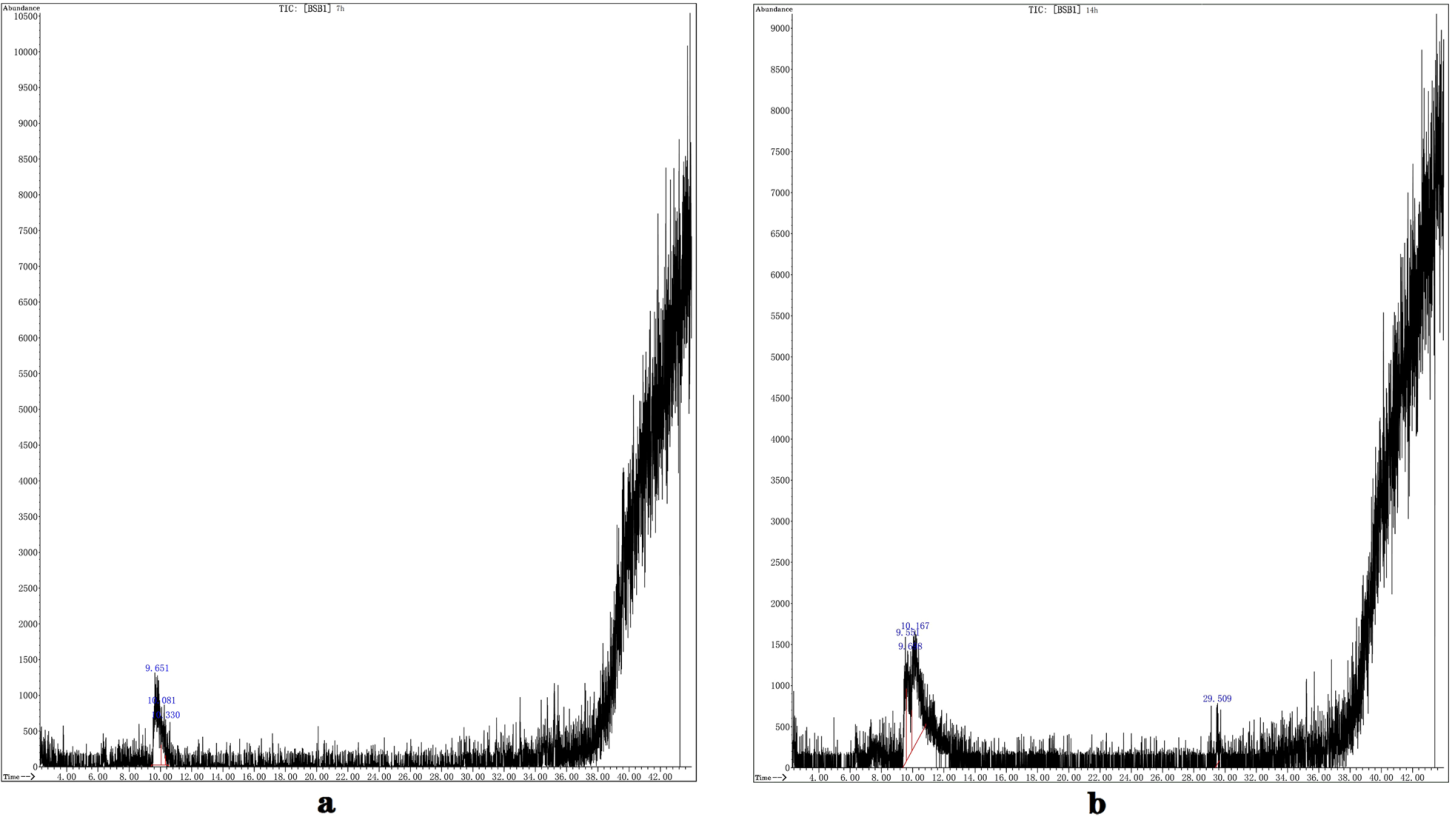
GC–MS Total ion chart of AR degradation by *T. gibbosa* at various stages. **a** 7 h GC–MS Total ion chart. **b** 14 h GC–MS Total ion chart

Using GC–MS methods, the degradation products can be more comprehensively analysed to supplement LC–MS results. It can be observed in the above total ion chromatography (Fig. 7a, b) that the abundance of material absorption ranges from 10,500 at 7 h to 9000 at 14 h, indicating that the pollutants that exist in the solution are decreasing. The detected substances in the GC–MS will be referenced to the NISETO mass spectrometry database for material matching. Intermediate products related to AR decomposition were detected as some small molecular substances (Table 4) such as 2- butene, acrylaldehyde and 1-butylene.

**Table 4:**
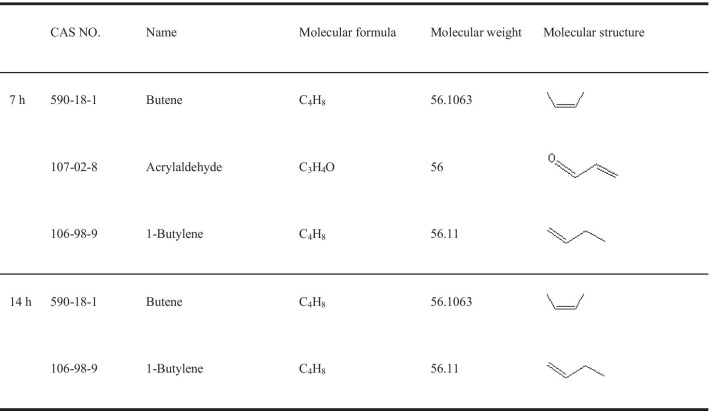
Relevant information of the intermediates by GC–MS

**Fig. 8:**
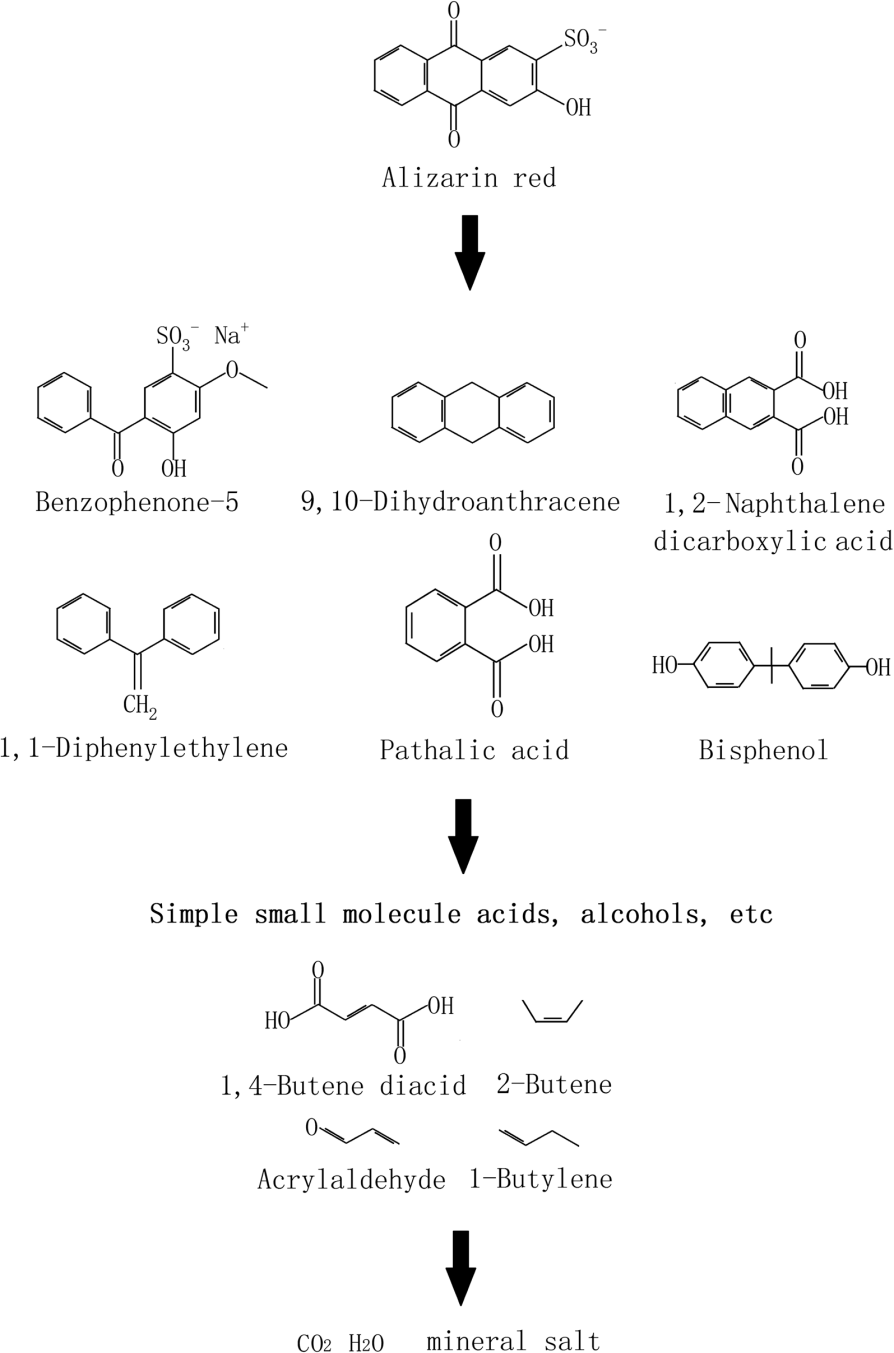
Extrapolation of AR degradation

The pathway of AR degradation was inferred from the analysis of *T. gibbosa* intermediate products of AR degradation by LC–MS and GC–MS (Fig. 8). The AR hydroxyl groups and sulfite roots were removed first, then the anthraquinone structure was broken, and finally, the benzene ring cracked to form small molecular inorganic salts. At this point, AR is completely degraded.

## Discussion

To understand the degradation of AR, the most studied aspects are chemical catalysis and physical adsorption. Hamza et al. performed the photocatalytic degradation of AR using a series of Cr-based manganese oxide nanomaterials (MH1–MH5). Overall, the AR decolorization rate was 70% [19]. The composite photocatalyst G-TiO_2_@Fe_3_O_4_ achieved 100% AR S to 100% at 60 min [20]. The active carbon fibers were degraded using the electrochemical oxidation method. The results showed that activated carbon fiber can effectively degrade the dye in wastewater via the anode electrochemical oxidation method, and the decolorization rate at 60 min can reach 98% [21]. The physical adsorbents commonly used in the industry include activated carbon, activated diatomite, natural peeling soil, fiber series, activation medium, and cinder [22, 23]. The adsorption of these substances was relatively efficient. Although chemical and physical methods work well, they both have some drawbacks. Therefore, the use of microorganisms easy to reproduce, strong adaptability, variable characteristics, research using external means, to create conducive to microbial growth, breeding conditions, through microbial enzymes to oxidation or restore dye molecules, destroy its unsaturated bonds, and hair color groups, can effectively degrade macromolecular organic pollutants to purify sewage. Several studies have reviewed the microbial decolorization and degradation of synthetic dyes [24]. For instance, Crini reviewed the decolorization and/or bioadsorption of various dyes in wastewater using (dead or living) biomass. Moreover, Ali described that fungi, bacteria, yeasts, and algae can efficiently degrade dyes. Recently, Mishra and Maiti summarized three major classes of reactive dyes (azo, anthraquinone, and triphenylmethane dyes) that can be degraded by various bacteria [25]. None of these reports have thoroughly studied the specific mechanisms of dye degradation. The discoloration effect of *Trametes gibbosa* on Sisoon reached only 20.21% after 14 h. We identified the key enzymes involved in AR degradation using transcriptional sequencing techniques and bioinformatics analysis. For example, MnP, Laccase, Lip and CYP450 has been widely studied in dye degradation, and GST which has been rarely studied in dye degradation. We inferred a mechanism of AR degradation by these enzymes under the action of these key enzymes. The relationship of intracellular and extracellular enzymes are connected in series.

For the present study, we aimed to examine the gene transcriptional changes of *T. gibbosa* and AR metabolites at different processing times. To determine the degradation of key AR genes and enzymes and the AR degradation process, a transcriptomic analysis of *T. gibbosa* with different AR processing times was performed for the first time of genes that encode enzymes from GST, MnP, laccase, lignin peroxidases, and VP. Of the oxidoreductase types, AR treatment resulted in higher expression levels in *T. gibbosa*. White rot fungi are by far the most efficient ligninolytic organisms described to date. This capability to degrade lignin is due to their extracellular nonspecific and nonstereoselective enzyme system is composed of laccase, lignin peroxidases and manganese peroxidases, which function together with H_2_O_2_-producing oxidases and secondary metabolites [26]. The same unique nonspecific mechanisms that give these fungi the ability to degrade lignin also allow them to degrade a wide range of pollutants. They are able to degrade polycyclic aromatic hydrocarbons (PAHs), chlorinated phenols, polychlorinated biphenyls, dioxins, pesticides, explosives and dyes [27, 28]. Purified laccases, lignin (LiP) and MnP are able to decolourize dyes of different chemical structures. It has been shown that these enzymes can degrade different dyes [29, 30]. In our study, the activity of MnP and laccase increased significantly after AR treatment. MnP, laccase, LiP and other genes were differentially expressed, and most of them were upregulated. In the studies of bacterial degradation of anthraquinone dyes it found that bacterial degradation of anthracinone dyes was due to a reduction reaction in which catalytic cracking of the conjugated dye bonds is performed by a reductase [31]. All the MnP, Laccase and LiP screened in this study belonged to oxidoreductase. This is the same as the bacterial degradation of anraquinone dyes. It can be inferred that oxidative reductases like MnP, Laccase and LiP of *T. gibbosa* may be involved in the catalytic cleavage of coupled dye bonds. This speculation remains to be verified by subsequent trials.

Since one of the basic functions of GST is detoxification, GST in metabolic aromatic compounds may be expressed in conjunction with degradation enzyme genes, so they may be used as reporter genes to monitor the expression of degradation pathway genes [32]. L Loyd-Jones et al. believe that genes encoding GST may be widely present in PAH-degrading bacteria and can be used as molecular tools for the detection of such bacteria. The authors obtained amplified bands from PAH-degrading bacteria isolated from sources in the United States, New Zealand and Antarctica. Successful amplification in contaminated soils also makes GST coding genes a useful tool for evaluating PAH-contaminated soils [33]. This study used transcriptome differential analysis to enrich the glutathione metabolic pathway (ko00480) in which GST genes are the most differentially enriched genes in this pathway, and most of them are upregulated. GST activity is also rising, which is consistent with previous studies.

In the study of bacterial degradation of anraquinone dyes, Andleeb et al. proposed that the first step of bacterial anthraquinone dye degradation involves the dissociation of the small molecular groups around the anthraquinone rings from the parent compound under aerobic conditions [34]. This was followed by a break to the anraquinone ring [35]. The anthraquinone ring is gradually broken, forming much smaller molecular compounds through oxidation and hydrolysis. However, based on the results of LC–MS and GC–MS in this study, I believe that small molecular groups around and anthracinone ring breaks for the degradation of anraquinone dyes may not have a sequential order. The two processes can be performed alternately.

1-amino-4-bromoanthraquinone-2-sulfonic acid degradation by *Sphingomonas herbicidovorans* FL, and deccattered down into phthalic acid, 2-amino-3-hydroxy-5-bromobenzenesulfonic acid or 2-amino-4-hydroxy- 5bromobenzenesulfonic acid [36]. Andleeb et al. analyzed the complete degradation pathway of Brilliant Blue K2RLby bacterial and found that the parent compound was initially broken at the attachment site of the reactive group, releasing the anthraquinone moiety [34]. Further cleavage of the anthraquinone metabolites led to the formation of small molecules such as benzoic acid. Chemical products with strong ionization potential degrade AR as 1,4,5,8-tetrahydroxyanthraquinone, 1,2-naphthalene dicarboxylic acid, adjacent hydroxybenzoic acid, phthalate, phthalic anhydride, bisphenol, 1,4-butyl phthalic acid, 2-oxybutyl acid, and butanol [37]. Through LC–MS and GC–MS, we obtained a richer variety of *T. gibbosa* products to degrade AR. Such as 1,4-butene diacid, phthalic acid, 1,1-diphenylethylene, 9,10-dihydroanthracene, 1,2-naphthalene dicarboxylic acid, bisphenol, benzophenone-5, and indicating more diverse pathways for fungi to degrade dyes. We also found that phthalic acid is an important intermediate in the degradation of anthracquinone dyes, both chemical degradation or biodegradation such as bacteria or fungi.

In future studies, we aim to obtain the efficient degradation of *T. gibbosa* through molecular-level techniques. The present study provides a theoretical basis for the application of dye wastewater degraded by white rot fungi.

## Conclusion

A transcriptome analysis of AR *T. gibbosa* showed the following: (1) The differentially expressed genes encoding oxidoreductase in the AR environment combined with the MnP and laccase activity results indicate that oxidoreductase is the key enzyme for AR degradation. (2) The glutathione metabolic pathway (ko00480) was screened, and binding GST activity and GSH content indicated that the glutathione metabolic pathway is involved in AR degradation. The AR degradation process was inferred by LC–MS and GC–MS, and 1,4- Butene diacid was the most intermediate. This study explored the process of AR biodegradation at the molecular and biochemical levels and provided a theoretical basis for its application in practical production.

## Supplementary Information

**Additional file 1. Table S1**: Sequencing and Assembly Statistics for the fifteen Transcriptome Data of *T. gibbosa* at Five Times of AR Treatment. **Table S2**: The number summarizes all annotated expressed genes. **Table S3**: The number of DEGs and the number summary of the annotated DEGs. **Table S4**: GO enrichment DEGs differential grouping. **Table S5**: DEGs from four important GO terms.

## Data Availability

The datasets used and/or analyzed during the current study are available from the corresponding author upon reasonable request. This study data was uploaded to the NCBI. NO.:PRJNA741348 (SRR15001034-SRR15001048).

## Abbreviations

*T. gibbosa*: *Trametes gibbosa*
AR: Alizarin red
MnP: Manganese peroxidase
LiP: Lignin peroxidase
Cytochrome P450: CYP450
GSH: Glutathione
GST: Glutathione S-transferase
TP: Total protein
VP: Versatile peroxidase
