# Supplementary material for: The mechanism of degradation of alizarin red by a white-rot fungus Trametes gibbosa
Source: BMC Biotechnol. 2021 Nov 5;21:64. doi: 10.1186/s12896-021-00720-8 (PMC8570020; doi:10.1186/s12896-021-00720-8)
Supplement: Supplementary file 1 — Additional file 1. Table S1: Sequencing and Assembly Statistics for the fifteen Transcriptome Data of T. gibbosa at Five Times of AR Treatment. Table S2: The number summarizes all annotated expressed genes. Table S3: The number of DEGs and the number summary of the annotated DEGs. Table S4: GO enrichment DEGs differential grouping. Table S5: DEGs from four important GO terms. [file 12896_2021_720_MOESM1_ESM.docx]

Table S1. Sequencing and assembly statistics for the fifteen transcriptome data of *T. gibbosa* at Five Times of AR Treatment.

| Time  Group | sample ID | No. of clean reads (×10^6^)^a^ | No. of clean bases (×10 ^9^) | GC Content  (%) | Q30  (%) | No. of mapped  reads (×10^6^) | Mapped  percentage (%) |
| --- | --- | --- | --- | --- | --- | --- | --- |
| 0 h | CK-1 | 22.37 | 6.69 | 59.98% | 92.74% | 39.67 | 88.65% |
|  | CK-2 | 20.65 | 6.16 | 60.22% | 93.16% | 36.90 | 89.33% |
|  | CK-3 | 21.78 | 6.51 | 60.29% | 93.12% | 38.97 | 89.48% |
| 3 h | QSH1-0 | 23.03 | 6.89 | 60.12% | 92.70% | 40.55 | 88.02% |
|  | QSH1-1 | 25.99 | 7.78 | 60.34% | 91.56% | 45.91 | 88.30% |
|  | QSH1-2 | 23.41 | 7.00 | 60.18% | 92.42% | 41.94 | 89.57% |
| 7 h | QSH2-0 | 22.49 | 6.73 | 60.29% | 92.58% | 39.24 | 87.22% |
|  | QSH2-1 | 26.44 | 7.91 | 60.27% | 91.32% | 46.75 | 88.40% |
|  | QSH2-2 | 25.97 | 7.77 | 60.30% | 91.96% | 46.38 | 89.31% |
| 10 h | QSH3-0 | 21.38 | 6.40 | 60.26% | 92.47% | 37.34 | 87.30% |
|  | QSH3-1 | 25.53 | 7.64 | 60.38% | 92.12% | 45.64 | 89.39% |
|  | QSH3-2 | 27.96 | 8.37 | 60.34% | 92.58% | 50.48 | 90.28% |
| 14 h | QSH4-0 | 24.40 | 7.30 | 60.22% | 92.35% | 43.77 | 89.68% |
|  | QSH4-1 | 23.58 | 7.05 | 60.35% | 92.70% | 42.44 | 90.01% |
|  | QSH4-2 | 30.37 | 9.09 | 60.13% | 93.25% | 55.47 | 91.32% |
|  | Total |  | 109.29 |  |  |  |  |
| a Here, no. is short for number. | | | | | | | |

Table S2. The number summary of all annotated expression genes.

| The annotated genes | | Total | Database | | | | | | | | Over all |
| --- | --- | --- | --- | --- | --- | --- | --- | --- | --- | --- | --- |
|  |  |  | GO | COG | KEGG | KOG | Pfam | Swiss Prot | eggNOG | NR |  |
| All genes | Number | 12921 | 5346 | 3648 | 3616 | 4947 | 7074 | 5602 | 8736 | 10551 | 10560 |
|  | Percentage (%) | 100 | 41.37 | 28.23 | 27.99 | 38.27 | 54.75 | 43.36 | 67.61 | 81.66 | 81.72 |

Table S3. The number of DEGs and the number summary of the annotated DEGs.

| Group | DEGs total | DEGs up | DEGs down | Annotation number | Annotation percentage(%) |
| --- | --- | --- | --- | --- | --- |
| CK vs QSH1 | 620 | 288 | 332 | 578 | 93.23 |
| CK vs QSH2 | 663 | 232 | 431 | 595 | 89.74 |
| CK vs QSH3 | 543 | 182 | 361 | 511 | 94.11 |
| CK vs QSH4 | 792 | 313 | 479 | 749 | 94.57 |
| QSH1vs QSH2 | 120 | 69 | 51 | 103 | 85.83 |
| QSH1vs QSH3 | 107 | 58 | 49 | 94 | 87.85 |
| QSH1vs QSH4 | 278 | 127 | 151 | 262 | 94.24 |
| QSH2vs QSH3 | 62 | 31 | 31 | 57 | 91.94 |
| QSH2vs QSH4 | 109 | 46 | 63 | 97 | 88.99 |
| QSH3vs QSH4 | 44 | 27 | 17 | 38 | 86.36 |
| Total | 1480 | -- | -- | 1370 | 92.57 |

Table S4 GO enrichment of DEGs in differential groups.

| CK vs QSH1 Up-expression DEGs | | | |
| --- | --- | --- | --- |
|  | GO Term | -log10(KS) | Gene |
| Biological Process | GO:0000041 transition metal ion transport | 2.66 | gene_1787,gene_356 |
|  | GO:0046274 lignin catabolic process | 2.52 | gene_3889,gene_3902 |
|  | GO:0006605 protein targeting | 2.43 | gene_1001,gene_5208,gene_5690 |
|  | GO:0006511 ubiquitin-dependent protein catabolic process | 2.40 | gene_1966,gene_3334,gene_3672,gene_7006,gene_963 |
|  | GO:0072594 establishment of protein localization to organelle | 2.28 | gene_1001,gene_5208,gene_5690 |
|  | GO:0000001 mitochondrion inheritance | 2.14 | gene_7356 |
|  | GO:0006696 ergosterol biosynthetic process | 2.14 | gene_10067 |
| Cellular Component | GO:0005741 mitochondrial outer membrane | 2.60 | gene_5690 |
| Molecular Function | GO:0020037 heme binding | 3.82 | gene_11537,gene_11851,gene_4,gene_4488,gene_5216,gene_7628,gene_8611,gene_9113 |
|  | GO:0004601 peroxidase activity | 2.51 | gene_11537,gene_11851,gene_4178,gene_8611 |
|  | GO:0003723 RNA binding | 2.42 | gene_1305,gene_4029,gene_8065,gene_8816,gene_9056,gene_963 |
|  | GO:0004298 threonine-type endopeptidase activity | 2.06 | gene_1966,gene_3334,gene_3672,gene_7006,gene_963,gene_965 |
| CK vs QSH1 Down-expression DEGs | | | |
| Biological Process | GO:0006536 glutamate metabolic process | 2.74 | gene_1202,gene_7547,gene_7548,gene_796 |
|  | GO:0035966 response to topologically incorrect protein | 2.29 | gene_2464,gene_6466 |
|  | GO:0006605 protein targeting | 2.23 | gene_5601,gene_688 |
|  | GO:0072594 establishment of protein localization to organelle | 2.09 | gene_5601,gene_688 |
|  | GO:0000001 mitochondrion inheritance | 2.05 | gene_3864 |
| Cellular Component | GO:0005741 mitochondrial outer membrane | 2.06 | gene_1180 |
| Molecular Function | GO:0020037 heme binding | 3.23 | gene_22,gene_4787,gene_713,gene_7522,gene_8119 |
|  | GO:0004521 endoribonuclease activity | 2.78 | gene_3969,gene_3993 |
|  | GO:0050660 flavin adenine dinucleotide binding | 2.19 | gene_1807,gene_22,gene_2875,gene_3556,gene_9842 |
|  | GO:0016620 oxidoreductase activity, acting on the aldehyde or oxo group of donors, NAD or NADP as acceptor | 2.17 | gene_1202,gene_7919 |
| QSH1 vs QSH4 Up-expression DEGs | | | |
| Biological Process | GO:0006605 protein targeting | 2.38 | gene_5601 |
|  | GO:0072594 establishment of protein localization to organelle | 2.24 | gene_5601 |
| Molecular Function | GO:0016620 oxidoreductase activity, acting on the aldehyde or oxo group of donors, NAD or NADP as acceptor | 2.49 | gene_3674,gene_7919 |
|  | GO:0020037 heme binding | 2.42 | gene_5139 |
|  | GO:0003723 RNA binding | 2.2 | gene_1305,gene_802 |
|  | GO:0004540 ribonuclease activity | 2.05 | gene_802 |
| QSH1 vs QSH4 Down-expression DEGs | | | |
| Biological Process | GO:0046274 lignin catabolic process | 2.64 | gene_3889,gene_3902 |
|  | GO:0072593 reactive oxygen species metabolic process | 2.40 | gene_7517 |
|  | GO:0006696 ergosterol biosynthetic process | 2.18 | gene_10067,gene_10936 |
|  | GO:0010035 response to inorganic substance | 2.12 | gene_4178,gene_7517 |
|  | GO:1901701 cellular response to oxygen-containing compound | 2.02 | gene_7517 |
| Cellular Component | GO:0005741 mitochondrial outer membrane | 2.70 | gene_10936,gene_5809 |
| Molecular Function | GO:0020037 heme binding | 3.37 | gene_4,gene_4487,gene_5216,gene_5570 |
|  | GO:0004601 peroxidase activity | 2.21 | gene_274,gene_4178 |
| Note: KS: the significant statistics of the enrichment of the GO category, the smaller the KS value is the larger the log10(KS) is, indicating the more significant the enrichment. | | | |

Table S5 DEGs from four important GO terms.

| GO term | Gene function | Gene No. |
| --- | --- | --- |
| Heme binding (GO:0020037) | 4-hydroxysphinganine ceramide fatty acyl 2-hydroxylase | gene_5570 |
|  | Versatile peroxidase | gene_11851, _11537, _713 |
|  | MnP | gene_8611 |
|  | Acyl-CoA dehydrogenase | gene_22, _4 |
|  | Cytochrome P450 | gene_7628,_9113,_4488,_5216,_11472,_4487, gene_5139,_7522,_10935,_6568 |
|  | Fumarate reductase | gene_8119 |
|  | L-lactate dehydrogenase (cytochrome) | gene_4787 |
|  | nitric oxide dioxygenase | gene_10651 |
| Lignin catabolic (GO:0046274) | Laccase | gene_3889,_3902,_1741 |
| oxidoreductase activity, acting on the aldehyde or oxo group of donors, NAD or NADP as the acceptor (GO:0046274) | 1-pyrroline-5-carboxylate dehydrogenase | gene_1202 |
|  | aldehyde dehydrogenase (NAD+) | gene_3674 |
| Peroxidase activity (GO:0004601) | Versatile peroxidase | gene_11851,_11537, _713 |
|  | MnP | gene_8611 |
